# The enhanced recovery after surgery (ERAS) protocol to promote recovery following esophageal cancer resection

**DOI:** 10.1007/s00595-020-01956-1

**Published:** 2020-02-11

**Authors:** Apurva Ashok, Devayani Niyogi, Priya Ranganathan, Sandeep Tandon, Maheema Bhaskar, George Karimundackal, Sabita Jiwnani, Madhavi Shetmahajan, C. S. Pramesh

**Affiliations:** 1 Division of Thoracic Surgery, Department of Surgical Oncology, Tata Memorial Centre, Tata Memorial Hospital, Homi Bhabha National Institute, Parel, Mumbai, 400012 India; 2 Division of Thoracic Surgery, Department of Anesthesiology, Critical Care and Pain, Tata Memorial Centre, Homi Bhabha National Institute, Mumbai, India; 3 Division of Thoracic Surgery, Department of Pulmonary Medicine, Tata Memorial Centre, Homi Bhabha National Institute, Mumbai, India

**Keywords:** Esophageal surgery, Esophagectomy, Enhanced recovery

## Abstract

Esophageal cancer surgery, comprising esophagectomy with radical lymphadenectomy, is a complex procedure associated with considerable morbidity and mortality. The enhanced recovery after surgery (ERAS) protocol which aims to improve perioperative care, minimize complications, and accelerate recovery is showing promise for achieving better perioperative outcomes. ERAS is a multimodal approach that has been reported to shorten the length of hospital stay, reduce surgical stress response, decrease morbidity, and expedite recovery. While ERAS components straddle preoperative, intraoperative, and postoperative periods, they need to be seen in continuum and not as isolated elements. In this review, we elaborate on the components of an ERAS protocol after esophagectomy including preoperative nutrition, prehabilitation, counselling, smoking and alcohol cessation, cardiopulmonary evaluation, surgical technique, anaesthetic management, intra- and postoperative fluid management and pain relief, mobilization and physiotherapy, enteral and oral feeding, removal of drains, and several other components. We also share our own institutional protocol for ERAS following esophageal resections.

## Introduction

Esophageal cancer surgery is a major and complex surgery comprised of esophagectomy with radical lymphadenectomy. It is still associated with unacceptable morbidity and mortality rates. A worldwide review from high-volume centres performing esophagectomy showed overall morbidity of 59% and 30-day mortality of 2.4% [1]; however, national audits have recorded higher 30-day mortality after esophagectomy of around 5% and 90-day mortality of 13% [2]. With these outcomes, we must endeavour to reduce complications and promote early recovery. One such strategy showing promise is the enhanced recovery after surgery (ERAS) protocol, which aims to improve perioperative care, minimize complications, and accelerate recovery.

The concept of ERAS was first described by Henrik Kehlet in 1997, in the setting of colorectal surgery [3, 4]. It has evolved over the years into a multidisciplinary team approach involving surgeons, anaesthesiologists, critical care physicians, physiotherapists, nutritionists and nurses in the perioperative care of the patient and integrating evidence-based protocols into clinical practice. This multimodal approach has been shown to shorten the length of hospital stay, reduce surgical stress response, decrease morbidity, and expedite recovery [5]. Subsequently, the ERAS society was established in 2010 and guidelines have been published for colorectal, bariatric surgery, gastrectomy, liver surgery and gynaecologic oncology. The implementation of ERAS protocols has decreased the cost of overall treatment without compromising outcomes [6].

Evidence for using the ERAS protocol after esophageal surgery is limited. Studies addressing the feasibility of ERAS after esophagectomy have investigated various protocols with different components of ERAS as there were no standardized guidelines until 2018. One of the earlier systematic reviews in 2014 [7], analyzing retrospective studies using a non-standardized ERAS protocol, reported favourable morbidity, mortality, and length of stay but concluded that the evidence was weak and incomplete. A subsequent meta-analysis by Pisarska et al. [8], of 2042 patients (1058 in an ERAS group vs 984 in a traditional group), revealed a significantly shorter hospital stay and fewer non-surgical and pulmonary complications in the ERAS group, but no effect on overall morbidity, mortality or readmission rates. Another meta-analysis [9] identified factors that could form the core components of ERAS for esophageal surgery specifically. Standardization of the ERAS protocol should become easier after the recent publication by the ERAS society of the guidelines for perioperative care in esophagectomy [10]. The current guidelines are proposed to specifically improve surgical outcomes after esophageal resection with interventions in all three phases: preoperative, intraoperative, and postoperative. Table 1 summarizes the key components of the ERAS protocol.

**Table 1:** Components of the enhanced recovery after surgery (ERAS) protocol

Preoperative	Intraoperative	Postoperative
Preoperative nutrition Prehabilitation Patient education and counselling Smoking and alcohol cessation Multidisciplinary team Cardiopulmonary assessment Venous thrombo-prophylaxis Preoperative fasting and carbohydrate-rich loading	Surgical approach Anaesthetic management Perioperative fluid management Prevention of hypothermia	Early mobilization Early removal of drains Early enteral feeding Perioperative pain control Postoperative nausea and vomiting Postoperative glycemic control

## ERAS components

The preoperative components aim to optimize and prepare the patient for surgery; the intraoperative elements, consisting of surgical and anaesthesia techniques, aim to minimize disruption of physiology, and the postoperative components aim to promote patient rehabilitation and recovery. However, while this classification enables enlisting individual components, the separation is largely artificial and the ideal ERAS protocol should flow seamlessly between these three phases.

## Preoperative strategies

### Preoperative nutritional assessment and intervention (Table 2)

**Table 2:** Nutritional risk assessment and intervention based on the ESPEN guidelines [10]

Nutritional risk		Intervention
Low risk	Normal intake Minimal weight loss	Dietary advice
Moderate risk	Anorexia/dysphagia Unintentional weight loss 5–9%	Protein and energy supplements
High risk	Severe dysphagia Unintentional weight loss > 10% Body mass index < 18 kg/m^2^	Enteral support (tube feeds)

Patients with esophageal cancer have a high prevalence of malnutrition because of the dysphagia caused by the tumor, with secondary anorexia and cancer cachexia. Significant weight loss and nutritional deficiencies predispose to an increased risk of complications and protracted admission [11]. Sarcopenia and frailty from cancer cachexia also impact on patient outcomes and recovery [12, 13]. Hence, it is essential to assess the nutritional status of the patient at diagnosis. The decision to intervene is based on risk assessment, but nutritional supplementation is known to have a positive effect on perioperative outcomes [14, 15].

The role of pharmaco-nutrition or immune nutrition in reducing oxidative stress and inflammatory response, and thereby improving postoperative morbidity, has been demonstrated in studies on gastrointestinal cancer [16]. However, there is limited information about administering immune-stimulating nutrients such as omega-3 fatty acids, arginine or nucleotides to esophageal cancer patients [17]. Esophageal cancer patients are often anemic and may require blood transfusion, which in turn can impact morbidity [18]. Evidence regarding the use of iron or erythropoietin to correct anemia in these patients is limited [19].

### Prehabilitation programs

A patient’s initial physiological reserve has a bearing on the return of functional activity after a major surgical procedure. Hence, efforts to optimize functional capacity prior to surgery have shown improved outcomes for some abdominal and oncologic surgeries [20]. A multimodal approach termed as ‘prehabilitation’ comprising nutritional supplementation, psychological counselling, medical optimization, and a structured exercise program involving both aerobic and strengthening activity has been proposed to improve outcomes [21]. Respiratory optimization with deep breathing exercises, spirometry, and inspiratory muscle training decreases pulmonary complications [22]. Studies have shown that prehabilitation reduces anxiety, depression and fatigue, and improves the quality of life [23]. Preliminary results from a recently concluded study [24] on prehabilitation for esophageal surgery demonstrated improvement in functional capacity; however, the impact on postoperative outcomes needs to be evaluated by further ongoing studies [25]. Extrapolation from studies on other abdominal surgeries [20] suggests that at least 4 weeks of preoperative prehabilitation is better than postoperative rehabilitation to influence outcomes. As most patients with operable esophageal cancer would receive neoadjuvant treatment, it would be opportunistic to use this preoperative window to initiate prehabilitation.

### Patient education/counselling

Preoperative structured counselling is essential to prepare patients for surgery, and reduce anxiety and confusion. Most ERAS guidelines incorporate patient education as an integral component and have online material for pre-esophagectomy education [26]. Some studies have shown alleviation of anxiety and improved retention of information with detailed counselling [27]. Hence, the recommendation is strong for preoperative education with an emphasis on what to expect and clarification of postoperative targets.

### Smoking and alcohol cessation

The importance of smoking and alcohol cessation should also be emphasized in the counselling session with the patient and care-givers. Cessation of smoking for at least 1 month has been shown to reduce postoperative complications, significantly, especially pneumonia and wound infections [28, 29]. Similarly, patients who consume alcohol regularly have increased cardiopulmonary and hemorrhagic morbidity, which has been seen to decrease with 4 weeks of abstinence [30].

### Cardiopulmonary assessment

Cardiac and pulmonary status is assessed routinely before major surgery such as esophagectomy, to identify patients requiring preoperative optimization and those at higher risk of postoperative complications. Routine tests comprising echocardiography, spirometry, and a pulmonary function test (PFT) with diffusion lung capacity are done to assess cardiac and pulmonary status separately. Cardiopulmonary exercise testing (CPET) provides an integrated assessment of the body’s response to stress and is a reliable tool to stratify patients at risk of major complications after elective surgery [31, 32]. Few studies have evaluated the role of CPET in patients undergoing esophagectomy and the results have been inconclusive [33–37]. Further research is needed to establish the role of CPET as a preoperative assessment tool for patients undergoing esophagectomy. Sub-optimal tests like the 6-min walk test and the shuttle walk test have been used as substitutes for a formal cardiopulmonary exercise test, but there are limited data to validate their role in the risk stratification of patients undergoing esophagectomy [34–36]. In our practice, only patients with significant cardiopulmonary risk factors are subjected to a CPET.

### Multidisciplinary tumor board/multidisciplinary pathway

A multidisciplinary team (MDT) of specialist professionals improves the quality of care delivered. Esophageal cancer studies have demonstrated more accurate staging, better treatment selection, and improved outcomes when decisions were made by a tumor board [37]. Hence, it is recommended that all patients are evaluated and managed by an MDT. During the perioperative period, a multidisciplinary clinical pathway utilizing the skills of professional specialists has been shown to benefit patient care and outcomes [38].

### Venous thrombo-prophylaxis

The incidence of venous thromboembolism after esophagectomy ranges from 5 to 7%, with a doubled risk of mortality [39]. The current American College of Chest Physicians Guidelines recommend a combination of chemical (low molecular weight heparin or unfractionated heparin) and mechanical (elastic stockings or pneumatic compression devices) treatment for adequate prophylaxis in high-risk patients. They also recommend that chemoprophylaxis be started 2–12 h before surgery and continued for 4 weeks after [40, 41].

### Fasting and carbohydrate loading

The American Society of Anesthesiologists practice guidelines for preoperative fasting recommend allowing clear fluids to patients undergoing elective surgery, who are not at increased risk of aspiration, up until 2 h prior to surgery [42]. Patients undergoing esophagectomy may have varying degrees of obstruction and dysphagia; therefore, these guidelines should be individualized carefully. Preoperative carbohydrate loading is an integral part of the ERAS pathway and has been shown to positively influence several markers of perioperative outcomes, but specific data for esophagectomy are lacking [43].

## Intraoperative strategies

### Surgical management

#### Timing of surgery after neoadjuvant therapy

Neoadjuvant therapy, being either chemotherapy or chemoradiation, is part of the standard of care for stage II and resectable stage III esophageal cancer [44]. Both forms of neoadjuvant therapy suppress immunity and healing. Surgery needs to be scheduled after an adequate interval following chemotherapy and/or radiation. In the FLOT 4 trial, surgery was scheduled for 4 weeks from the last dose of chemotherapy [45]. The CROSS trial, which laid the basis for neoadjuvant chemoradiation for esophageal cancer, recommended an interval of 4–6 weeks from the last date of radiotherapy [46]. Extrapolating from rectal cancer, some investigators believe that increasing the time to surgery improved the rate of pathological complete response, while others believe that the resulting increased fibrosis made dissection more difficult [47, 48]. A study by Kim et al. concluded that delaying surgery after chemoradiation did not improve complete response rates or survival [49]. The ERAS Society guidelines suggest 3–6 weeks after chemotherapy and 6–8 weeks after the last day of radiotherapy as the optimum timing for surgery [10].

#### Type of surgery

The level of disease on endoscopy, cross-sectional imaging, and histology, as well as the patient’s general fitness, determine the choice of surgery for esophageal cancer. According to a meta-analysis by Hulscher et al. [50], transhiatal esophagectomy was associated with fewer pulmonary complications, but trans-transthoracic esophagectomy showed a trend towards improved survival. A subsequent subgroup analysis based on the location of the primary tumor showed better survival of patients after transthoracic resection and with a limited number of positive lymph nodes [51]. All of the three meta-analyses performed to compare transhiatal esophagectomy versus transthoracic esophagectomy to date have been based largely on retrospective studies [52, 53]. A transthoracic resection offers the best chance for complete resection with negative margins, accurate lymphadenectomy and staging, and possibly better loco-regional control and survival. Therefore, at our centre, all patients preferentially undergo trans-thoracic esophagectomy. Transhiatal esophagectomy is considered only for patients with compromised pulmonary function, extensive pulmonary fibrosis, or borderline fitness. For patients with stomach involvement precluding an adequate length of stomach tube to reach the neck, an Ivor Lewis approach can be considered, with the anastomosis in the chest. For Siewerts 3 GE junction adenocarcinoma, evidence also supports the use of a left thoraco-abdominal approach, provided adequate margins can be achieved [54].

#### Minimally invasive surgery

Esophagectomy can be performed via open, video-assisted thoracoscopy and laparoscopy, robotic, or hybrid techniques. Long-term follow-up of the randomized TIME trial showed that minimally invasive esophagectomy is associated with less blood loss, shorter hospital stay, fewer pulmonary infections, better quality of life at 1 year, and equivalent oncological outcomes at 3 years [55]. The recently published MIRO trial also found better short-term outcomes of the minimally invasive approach with no compromise in surgical quality [56]. Robotic resections have shown equivalent long-term outcomes in several series [57] with a randomised trial observing fewer complications than after the open approach [58]. However, their superiority over the VATS approach is still uncertain. The minimally invasive approach for esophagectomy is in keeping with the principles of ERAS and has shown superior short-term outcomes without compromising oncological efficacy in several meta-analyses [59–61]. It is, therefore, recommended when feasible.

#### Extent of lymphadenectomy

In the absence of involved supra-carinal lymph nodes, a two-field (infra-carinal mediastinal and D2 abdominal) lymphadenectomy is standard for esophageal cancer [44]. A study evaluating the Worldwide Esophageal Cancer Collaboration (WECC) database suggested that a greater number of lymph nodes retrieved were associated with better survival and recommended lymphadenectomy according to the T stage [62]. Three-field lymphadenectomy, including supra-carinal mediastinal and bilateral recurrent laryngeal nodal chains, has shown survival benefit, especially for patients with squamous esophageal cancer [63, 64]. A randomized trial comparing the standard two-field with radical three-field lymphadenectomy for operable esophageal cancer is currently underway at our institution (NCT 00193817). Three-field lymphadenectomy involves extensive handling of bilateral recurrent laryngeal nerves and is associated with an increased incidence of vocal cord palsy and anastomotic leak. This has a direct impact on the patients’ postoperative course. However, in most studies, postoperative mortality is similar in the two groups [64, 65]. We think that the choice of lymphadenectomy should be dictated more by oncological principles than by the principles of ERAS.

#### Choice of conduit

The stomach, jejunum, and colon are options to use for reconstructing the esophagus after esophagectomy [66]. Of these, the stomach, being easy to mobilize into the posterior mediastinum and with its robust vascular supply, is usually the conduit of choice. Using the stomach for reconstruction also has the advantage of only one anastomosis. Fashioning a gastric tube rather than using the entire stomach is associated with a reduced risk of delayed gastric emptying and bile reflux, and a better quality of life [67]. In certain lesions where the extent of stomach involvement precludes its use as a conduit, the jejunum or colon might have to be used. A colonic conduit needs preoperative imaging for vascular supply, a colonoscopy to rule out any pathology, and adequate bowel preparation prior to surgery [66]. Using the colon increases operative time and involves three gastro-intestinal anastomoses. For a jejunal conduit to reach the neck, super-charging with microvascular anastomosis is required, making it technically challenging [10]. While recourse to the colon as esophageal replacement may be mandated by extensive gastric involvement, using a gastric tube for replacement would be preferable in most other situations [66].

#### Role of pyloroplasty

Esophagectomy is typically associated with a vagotomy that denervated the pylorus, leading to pylorospasm. This might result in gastroparesis or gastric tube dilatation, which may increase the risk of aspiration. The role of a pyloric drainage procedure is controversial, but may be achieved by a pyloromyotomy, pyloroplasty, finger fracture, or internal dilatation. A 2002 meta-analysis concluded that although pyloric drainage decreased the incidence of outlet obstruction and fatal aspiration, it made no difference in terms of anastomotic leaks or pulmonary complications [68]. There is currently insufficient evidence to recommend any specific pyloric drainage procedure and it is generally left to the surgeon’s discretion.

#### Prophylactic thoracic duct ligation

The incidence of chylothorax after trans-thoracic esophagectomy ranges from 0.6 to 4% [69]. Persistent chylothorax leads to hypovolemia, immune suppression, metabolic disturbances, nutritional depletion, and sepsis, significantly increasing morbidity and sometimes mortality. Some studies have shown that mass ligation of all tissue between the aorta and azygous vein after thoracic dissection of the esophagus decreases the incidence of chyle leak significantly [70], but other studies show no difference [71]. In our institution, the thoracic duct is ligated only if it has been injured intraoperatively or dissected extensively.

#### Nasogastric decompression

A nasogastric tube placed within the gastric conduit across the anastomosis post-esophagectomy decompresses the stomach tube, and is preferred by most esophageal surgeons. Studies, including one conducted in our institution, have shown that continued nasogastric drainage beyond the second postoperative day does not decrease the incidence of anastomotic leaks or pulmonary complications [7, 72]. The ERAS society guidelines recommend routine nasogastric decompression post-esophagectomy, but with early removal, on day 2, when clinically appropriate [10].

#### Feeding access

Enteral feeding may be achieved via a naso-jejunal tube or a feeding jejunostomy placed intraoperatively or postoperatively. A surgically placed feeding jejunostomy has a 0.5% mortality rate and up to a 2.5% morbidity rate. Insertion site infection, dislodgement, and leakage are the most common complications. In comparison, naso-jejunal tubes add to patient discomfort, but have a much lower complication rate [73]. The ERAS society guidelines emphasize the use of an enteral feeding access route. The choice of route is left to the discretion of the surgeon [10].

### Anesthesia management

#### Ventilation strategies

Patients undergoing esophagectomy are prone to respiratory complications and the incidence of pulmonary morbidity is as high as 25% [74]. The focus of intraoperative ventilation is to minimize pulmonary trauma. Studies of patients undergoing major abdominal surgery have investigated the role of lung-protective ventilation and the use of positive end-expiratory pressure (PEEP) with conflicting results [72, 75]. In patients undergoing esophagectomy, randomized trials have shown that lung-protective ventilation reduces lung inflammation and pulmonary complications [76, 77]. The ERAS guidelines for the management of patients undergoing esophagectomy recommend the use of lung-protective strategies [10]. Although it has been postulated that pressure-controlled modes of ventilation may be superior to volume-controlled modes during one-lung ventilation; there are insufficient data to confirm this [78, 79].

#### Perioperative fluid management

The incidence of pulmonary complications after esophagectomy is high and this can be compounded by fluid overload, which leads to pulmonary interstitial edema. Several studies have shown that a higher cumulative fluid balance is associated with increased pulmonary morbidity in esophagectomy patients [80–84]. There is increasing emphasis on perioperative fluid restriction; however, a recent trial in patients undergoing major abdominal surgery compared liberal versus restrictive fluid regimens and found that there was no difference in the incidence of pulmonary complications, septic complications, or death, although the restrictive group had a significantly higher incidence of acute kidney injury. Patients undergoing esophagectomy have several specific problems: first, esophagectomy involves prolonged surgery, third-spacing, evaporative losses, and blood loss; therefore, an excessively restrictive strategy that does not account for these losses may lead to hypovolemia and related-complications. Second, most restrictive strategies depend on the use of goal-directed therapy with invasive arterial monitoring (pulse pressure variation), cardiac output monitoring (stroke volume variation) or esophageal Doppler (aortic blood flow). The utility of PPV and SVV in patients with an open thorax on low-tidal volume ventilation is unclear [85, 86]. Moreover, restrictive fluid regimens rely on the use of vasopressors to maintain perfusion pressure in the absence of hypovolemia. For patients undergoing esophagectomy, the perfusion of the gastric conduit depends on the right gastro-epiploic artery and there may be concerns that vasoconstriction can adversely affect flow to the gastric conduit. However, these fears seem unfounded and two small studies have shown that the use of vasopressors to counter the hypotension caused by TEA improves blood flow in the gastric conduit [87, 88]. The ERAS guidelines recommend ‘optimal’ fluid therapy using balanced crystalloids aiming for a weight gain of not more than 2 kg/day [10]

#### Prevention of hypothermia

Patients undergoing esophagectomy are at increased risk of perioperative hypothermia caused by prolonged intra-cavity surgery. Hypothermia (defined as a core temperature below 36 °C) can adversely affect drug metabolism and recovery from anaesthesia, increase coagulopathy and transfusion requirements, and cause patient discomfort. Hypothermia has also been identified as a risk factor for surgical site infections and cardiac complications [86, 89]. The ERAS guidelines recommend the use of multi-modal techniques such as forced air warming and fluid warming to prevent hypothermia [10, 89].

## Postoperative strategies

### Extubation and the intensive care unit

Newer methods of adequate pain relief, minimally invasive surgery, and judicious intraoperative fluid administration have made immediate extubation after esophageal surgery the norm rather than the exception for low-risk patients [90]. High-risk patients, patients on pressor support, and those who are hypothermic may be electively ventilated. After extubation, low-risk patients can be managed in a high dependency unit as the intensive care unit should be reserved for high-risk patients requiring intensive monitoring and support [91].

### Early mobilization and chest physiotherapy

Early, structured mobilization is an integral part of all ERAS guidelines [10]. Prolonged bed rest after surgery leads to muscle loss, increased pulmonary complications, insulin resistance, and increased risk of venous thromboembolism [92]. To circumvent these problems, patients should be encouraged to ambulate early in the postoperative period, preferably on the day of surgery. Incremental goals should be set in a structured pattern for each postoperative day according to the clinical status of the patient. Incentive spirometry should be taught during prehabilitation for effective use in the postoperative period. Active involvement of the chest physiotherapist makes early and goal-directed mobilization possible and effective [93].

### Perioperative analgesia

Thoracic epidural analgesia (TEA) has been shown to reduce pulmonary morbidity in patients undergoing major surgery. Accordingly, in patients undergoing esophagectomy, the use of TEA results in decreased pulmonary infections, chronic post-thoracotomy pain and postoperative mortality [94–97]. TEA has also been shown to decrease anastomotic leak rates, possibly by improving microcirculation [98, 99]. Guidelines for the management of post-thoracotomy pain emphasize the use of TEA as the first-line technique for postoperative analgesia [100]. Paravertebral blockade has been suggested as an alternative to TEA for acute thoracotomy pain, with equivalent analgesia and a better side-effect profile [101–104]. Current guidelines (ERAS) continue to emphasise the role of TEA along with a multi-modal approach for post-esophagectomy pain [10]. The role of adjuncts such as gabapentinoids, magnesium, lignocaine, and ketamine is still not well established. Our practice is to use TEA with a combination of local anaesthetic and an opioid, along with systemic acetaminophen and diclofenac. Eligible patients receive a single dose of pregabalin 12 h prior to surgery. Patients with ineffective TEA are offered opioid-based intravenous patient-controlled analgesia.

### Early enteral feeding

Many patients with esophageal cancer are nutritionally debilitated, even at diagnosis, related to varying periods of dysphagia. Early initiation of nutritional support postoperatively is important to maintain the status achieved with prehabilitation and to prevent postoperative complications related to malnutrition. Enteral nutrition supersedes parenteral nutrition after esophageal resection. Parenteral nutrition is associated with an increased incidence of metabolic disturbances, raised liver enzymes, and sepsis. It should be used only when enteral feeding is not feasible. Enteral nutrition should be started through the naso-jejunal tube or feeding jejunostomy on postoperative day (POD) 1. Feed volumes should be escalated depending on the patients’ tolerance, meeting calorie requirements by POD 3 [10].

### Early removal of tubes and drains

Placement of peri-anastomotic drains (neck or thorax) has not been conclusively shown to increase the detection of leak and, hence, is not routinely recommended [10, 105, 106]. A chest drain is placed routinely after esophagectomy to ensure lung expansion and to detect bleeding, air, chyle, and anastomotic leak. The ERAS society guidelines recommend the use of a single centrally placed chest drain [10]^.^

An esophagectomy patient typically has a naso-gastric tube, a nasojejunal or jejunostomy tube, an arterial line, an intravenous catheter, an epidural catheter, a chest tube, and a urinary catheter in the immediate postoperative period. These make early mobilization cumbersome. The nasogastric tube should be removed by POD 2 if the gastric tube is not dilated [107]. The urinary catheter can be removed after 48 h or once the diuretic phase is reached; however, removal of the urinary catheter while the epidural catheter is still in place carries a 26% chance of re-insertion, especially in elderly males [108]. Monitoring for urinary retention after catheter removal becomes important. Chest drains are associated with pain and immobilization [109]. Most centres remove chest drains when their output is 100–150 ml/day although there are no data to support this value [110]. In our institution, we remove chest drains at a threshold of 5 ml/kg body weight. In practice, the chest drain can be removed after confirming complete lung expansion on the chest X-ray, in the absence of air or chyle leak, generally by POD 2 [10].

### Oral feeding

Randomized trials assessing early oral intake after upper gastrointestinal resections have shown no benefit in delaying oral intake beyond 48 h. Early oral intake was associated with earlier discharge and fewer complications, but no separate analysis was presented for esophageal anastomoses, which present unique challenges [111, 112]. Potential modalities to ascertain anastomotic integrity include endoscopy, contrast swallow and computed tomography (CT) scan with oral contrast. There is inadequate evidence to justify the routine use of any modality prior to starting oral intake or to establish one modality as superior [7]. At our institution, we rely entirely on clinical grounds to start oral feeding.

A recent study evaluating the early institution of oral intake as liquids on POD 1 and semi-solids on POD 2 after esophagectomy found no increase in complications and an earlier return of bowel function with improved short-term quality of life with early oral intake. In this study, all patients underwent an endoscopic examination on POD 1 to test vocal cord function prior to any oral intake [113]. For patients with recurrent laryngeal nerve palsy and those at high risk for aspiration, evaluation of the swallowing function with video-endoscopy or video-fluoroscopy is recommended before initiating oral feeding [114]. Supervised swallowing rehabilitation, consisting of direct and indirect exercises, should be started while the patient is still in hospital [115].

## Our institutional protocol

Our institution in India is a high-volume cancer centre performing 180–200 esophageal cancer operations every year. Enhanced recovery components have been practised for the past 10 years. All patients with esophageal cancer are evaluated and managed by a MDT. Along with the staging investigations, cardiac and pulmonary assessments are done, using echocardiography and PFT with diffusion lung capacity. Functional capacity is assessed using stair climbing or a 6-min walk test. Most patients (95%) present with cancer in the locally advanced stage and are treated with neoadjuvant therapy. Simultaneously, they are assessed by a dietician for their nutritional status and the need for supplementation either orally or via enteral tube feeds. Prior to initiating neoadjuvant therapy, patients are started on a prehabilitation program with chest physiotherapy and spirometry, smoking cessation, and medical optimization, thereby using this window optimally. CPET is performed only if the routine tests (ECHO and PFT) reveal borderline functional capacity or in high-risk cases. Patients considered to be at high risk for surgery are discussed by a special MDT including pulmonologists, critical care physicians, thoracic anesthesiologists, and esophageal surgeons.

Patients undergo esophageal resection 4–6 weeks after neoadjuvant chemotherapy and 6–8 weeks following neoadjuvant chemoradiation. Antithrombotic prophylaxis with low molecular weight heparin is administered 12 h before surgery and continued until discharge. Prolonged fasting is avoided and patients are allowed clear liquids and carbohydrate-rich drinks until 2 h prior to esophagectomy. Minimally invasive surgery accounts for 40% of surgeries and the stomach is our preferred choice of conduit. We are currently recruiting patients who do not have enlarged supra-carinal lymph nodes on imaging to participate in a randomized trial comparing standard two-field with radical three-field lymphadenectomy (NCT 00193817). Two-field lymphadenectomy is performed for all middle and lower third tumors unless there is clinico-radiological evidence of nodes in the superior mediastinum or cervical region. In this subset, elective three-field lymphadenectomy is performed. We routinely dilate the pylorus internally and decompress the stomach with a nasogastric tube to prevent stasis, reflux and aspiration.

Most of our patients (including those undergoing minimally invasive surgery) receive thoracic epidural analgesia intra- and postoperatively. We use low tidal volumes (5–6 ml per kg body weight) during one-lung ventilation. All patients have invasive arterial pressure monitoring and fluid management is guided by intraoperative urine output, blood pressure, and serial lactate monitoring. Balanced crystalloids are used for maintenance, while colloids or blood may be used to replace blood loss. Cardiac output monitoring is reserved for patients with cardiac risk factors. We use forced air warming and fluid warmers to prevent hypothermia. More than 95% of our patients are extubated in the operating room, while postoperative ventilation is used only for those with hypothermia, hemodynamic instability or other specific problems.

Patients are mobilized on the evening of surgery. Early enteral feeding is started by POD 1 through a nasojejunal tube and oral sips are started by POD 4. The nasogastric tube is removed by POD 2 provided there is no gastric tube dilatation after a 12-h period of clamping it. No drains are used for the cervical anastomosis and chest drains are removed once their output decreases to < 5 ml/kg/day. The urinary catheter is removed on about POD 2, if urine output is adequate. Perioperative pain is controlled mainly by thoracic epidural analgesia and supplemented with Acetaminophen and NSAIDS. If pain relief with the epidural catheter is ineffective, patient-controlled analgesia with intravenous fentanyl is used.

During the entire perioperative period of patient care, a multidisciplinary team comprised of anaesthetists, physiotherapists, pulmonary physicians, dieticians, nurses, and surgeons work in conjunction to improve outcomes and facilitate early patient recovery. There is an ongoing study at our institute, evaluating adherence to the ERAS society recommendations. On analyzing our perioperative outcomes in two time periods: pre-ERAS(2001–2010) and post-ERAS (2011–2019), we found that there was a substantial reduction in overall morbidity and mortality rates (6.6% before 2010 vs 4.9% after 2011) with the introduction of ERAS (64% and 6.6% before 2010 vs. 43% and 4.9% after 2011, respectively).

## Conclusion

ERAS greatly improves the perioperative outcomes of esophageal surgery and reduces the length of stay in hospital. However, many of the ERAS society recommendations are based on low or moderate level of evidence, and need further evaluation and research. However, with the introduction of the standardized guidelines by the ERAS society, there is an opportunity to unify protocols worldwide, generate data, and make them comparable for analysis.
