# Correction to: The enhanced recovery after surgery (ERAS) protocol to promote recovery following esophageal cancer resection

**DOI:** 10.1007/s00595-020-01987-8

**Published:** 2020-03-25

**Authors:** Apurva Ashok, Devayani Niyogi, Priya Ranganathan, Sandeep Tandon, Maheema Bhaskar, George Karimundackal, Sabita Jiwnani, Madhavi Shetmahajan, C. S. Pramesh

**Affiliations:** 1 Division of Thoracic Surgery, Department of Surgical Oncology, Tata Memorial Centre, Tata Memorial Hospital, Homi Bhabha National Institute, Parel, Mumbai, 400012 India; 2 Division of Thoracic Surgery, Department of Anesthesiology, Critical Care and Pain, Tata Memorial Centre, Homi Bhabha National Institute, Mumbai, India; 3 Division of Thoracic Surgery, Department of Pulmonary Medicine, Tata Memorial Centre, Homi Bhabha National Institute, Mumbai, India

## Correction to: Surgery Today 10.1007/s00595-020-01956-1

The article The enhanced recovery after surgery (ERAS) protocol to promote recovery following esophageal cancer resection by Apurva Ashok, Devayani Niyogi, Priya Ranganathan, Sandeep Tandon, Maheema Bhaskar, George Karimundackal, Sabita Jiwnani, Madhavi Shetmahajan, and C. S. Pramesh was originally published electronically on the publisher's internet portal https://link.springer.com/article/10.1007/s00595-020-01956-1 on 11 February 2020 without Open Access.

With the author(s)' decision to opt for Open Choice, the copyright of the article changed on March 10, 2020 © The Author(s) 2020 and the article is forthwith distributed under the terms of the Creative Commons Attribution 4.0 International License (https://creativecommons.org/licenses/by/4.0/), which permits use, duplication, adaptation, distribution and reproduction in any medium or format, as long as you give appropriate credit to the original author(s) and the source, provide a link to the Creative Commons license, and indicate if changes were made.

The original article has been corrected.

